# Beyond the Scores: Gendered Interpretations of Emergency Medicine Resident Assessments of Interdependent Performances

**DOI:** 10.5334/pme.1937

**Published:** 2025-08-21

**Authors:** Asil El Galad, Stefanie S. Sebok-Syer, Michael Panza, Kristen Ng, Lorelei Lingard

**Affiliations:** 1 Department of Medicine, Michael DeGroote School of Medicine, McMaster University, Hamilton, Ontario, Canada; 2 Department of Emergency Medicine, Stanford University, Palo Alto, California, USA; 3 Department of Surgery, McMaster University, Hamilton, Ontario, Canada; 4 Department of Emergency Medicine, Cornell University, New York, USA; 5 Department of Medicine and Centre for Education Research and Innovation, Schulich School of Medicine & Dentistry, Western University, London, Ontario, Canada

## Abstract

**Purpose:**

In medicine, gender bias and gendered language within assessments of individual performance are well established. Recent shifts toward assessing interdependence (the ability to work supportively and collaboratively within teams) demand we understand how gender bias and gendered language influence assessments. In exploring how faculty assess residents’ interdependent performances, this study evaluated how gender-presentation influences faculty raters’ assessments of residents’ interdependence in Emergency Medicine (EM).

**Methods:**

Using a multiple-methods (an experimental within-subjects study with follow-up interviews), 18 EM faculty from Canada and the United States assessed scripted videos of identical clinical encounters acted by male- and female-presenting residents. Faculty assessed female residents via anonymous online surveys and, six months later, assessed male residents via follow-up interviews using the same clinical scenarios. After every clip, faculty completed entrustable professional activity (EPA) and Milestone ratings and provided narrative justifications. Statistical analyses were conducted using Wilcoxon signed-rank tests to assess gender differences in EPA and Milestone scores. Qualitative data were analyzed using thematic analysis to identify recurring, gendered patterns in narrative justifications.

**Results:**

Quantitative results revealed no gender differences in Milestone and EPA scores, except for the resuscitation entrustment rating, where male residents were rated less favorably (z = –3.09, p = 0.002). Qualitative findings uncovered subtle gender differences. For the same clinical performances, male residents were frequently described as leaders, while female residents as collaborative. Furthermore, male residents’ help-seeking was framed as proactive, whereas female residents’ help-seeking was indicative of lacking knowledge. Finally, bias was not consistent across genders: male leadership expectations could negatively flavor assessments of male collaborative performances.

**Conclusion:**

EPA and Milestone scores showed marginal gender-based differences, while narrative justifications reflected clear gendered expectations about residents’ interdependence. These findings highlight the need for equity-oriented assessment practices that interrogate both the numbers and the narratives. As team-based competencies like interdependence become central to clinical training, ensuring that assessments reflect fair, unbiased interpretations are essential to supporting all learners equitably.

## Introduction

Medical education programs use competency-based education and assessment models that rely on both numerical ratings and narrative judgements to evaluate resident performance. While much attention has been paid to scoring tools and frameworks, the interpretive layer of assessment—how behaviours are described, framed, and valued—remains less examined. This layer is particularly important, as bias can manifest not only in the scores residents receive, but also in the language and value ascribed to their actions. Research shows that characteristics such as gender presentation can influence assessments of competence and merit, threatening the validity of current evaluation frameworks [1,2]. For example, studies have found that reviewers expressed more doubt in recommendation letters written for female applicants than those written for their male counterparts [3]. While previous research has largely focused on numeric scores, fewer studies have explored how gender bias may shape the narrative framing of performance, despite emerging evidence of gender disparities in narrative feedback across high-stakes assessments in specialties such as Family Medicine, General Surgery, Internal Medicine, and Emergency Medicine (EM) [4,5,6,7]. Such patterns suggest that gendered expectations can influence both how competence is assessed and how it is interpreted. Yet, we know little about how gendered perceptions shape assessments in domains that are inherently interpersonal and interpretive, such as *interdependent* performance.

In this study, we define interdependence as a construct that captures the ways in which team members interact with one another—supportively (i.e., triggered by one team member’s insufficient expertise to perform within their scope of practice) and collaboratively (i.e., demanded from the recognition that high-quality patient care requires contributions from one’s team) [8]. This understanding stems from a conceptual advancement that foregrounds interdependence as a desired aspect of expert performance, rather than a novice state to be outgrown as competence develops [8,9]. However, unlike task-based competencies, interdependence is shaped by complex relational dynamics and is often inferred rather than directly observed, making it particularly susceptible to bias. Although we acknowledge that interdependence remains an evolving construct in medical education, it remains unclear whether it is characterized differently in assessments for male and female gender presentations. Studies highlight that physicians are frequently required to navigate and conform to gendered expectations during their clinical training, which we hypothesize influences differing perceptions of interdependence and can have implications for assessment. For instance, male physicians report lower levels of interdependent self-construal compared to female physicians [10,11,12,13]. In addition to the influence on professional roles and training environments, these gendered dynamics are evident in evaluative contexts: residency applications and letters of recommendation often emphasize leadership and innovation for male candidates, while highlighting communal traits such as teamwork and caring for female candidates [14,15,16,17]. Such patterns underscore how interdependence may be perceived, practiced, and assessed through a gendered lens.

EM provides a particularly relevant context for examining how interdependence is assessed. Despite efforts to improve equity, gender parity remains elusive in EM, and gender disparities in assessment have been previously documented [18,19,20,21,22]. While clinical work in EM is inherently interprofessional and team-based, its deep historical roots in the military continue to reinforce masculinized norms that emphasize traits like emotional control, detachment, and decisiveness—qualities shaped by EM’s origins in battlefield medicine where swift, autonomous action could determine patient outcomes [23,24]. These expectations continue to shape how residents are assessed [23,24]. For instance, a study examining qualitative differences in EM resident feedback found that faculty often described the ideal EM resident as embodying stereotypically masculine traits: a calm, confident, and decisive leader capable of managing high-pressure situations to ensure optimal outcomes—an image closely tied to EM’s military roots [25]. These historical and cultural ‘militarized’ norms related to EM may conflict with emerging expectations of interdependence and team-based collaboration, creating tensions in how residents’ behaviours are perceived and valued, particularly when filtered through a gendered lens. A recent scoping review by Mencheti et al. (2022) revealed that female EM residents more frequently received discordant feedback, containing higher rates of negative or critical comments compared to their male counterparts, suggesting that behaviours aligned with interdependence may be interpreted differently depending on gender presentation [26].

Despite the increasing relevance of interdependence, most validated assessment tools in EM (and other specialties) focus on group-level performance rather than individual interdependence [27,28,29,30,31]. Currently, assessments of “interdependence” are often inferred from broader teamwork ratings [31,32,33]. Tools such as the Ottawa Global Rating Scale, Multi-disciplinary Team Performance Assessment Tool, Nurse-Physician Collaboration Scale, and Jefferson Scale of Attitudes Toward Nurse-Physician Collaboration assess team dynamics or attitudes toward collaboration, but may not capture the nuanced ways that gender bias shapes faculty interpretation of interdependent behaviour [34,35,36,37,38].

While prior studies have documented gender-disparities in assessment scores, not all forms of bias manifest numerically [20,39,40,41]. Some emerge in how performances are described, or rationalized, rather than how they are rated. In this study, we sought to examine not only whether gender influenced assessment scores, but also whether it shaped the narrative justifications for those scores, particularly in the assessment of interdependent behaviours. We analyzed faculty ratings and narrative comments within assessment tools not originally designed to measure interdependence—Milestones and Entrustable Professional Activities (EPAs)—but that capture team-based behaviours. These tools served as proxies to infer interdependence and illuminate how assessors interpret such behaviours. Although no widely accepted instrument currently exists to directly evaluate interdependence, our goal was to map how assessors interpret interdependent performances to evaluate the interpretive space in which they operate. By doing so, we aim to highlight gaps in existing assessment systems and inform the development of more precise tools that reflect the complex, interdependent nature of clinical work.

Although our study is limited to binary gender presentation (male- and female-presenting residents), this choice was made to reduce ambiguity in rater perceptions and maintain methodological clarity. We recognize the term gender refers to socially constructed roles, behaviors, and identities that are not binary or static, and future work must explore how non-binary and gender-diverse presentations are interpreted in assessment.

## Method

This study received ethical approval from our institution’s IRB (#116677).

Using a knowledge-generating evaluation framework, which focuses on understanding the general effectiveness of a program or project and highlights lessons learned to inform design and practice, this multiple-methods study included quantitative and qualitative data from one-group of faculty participants, more specifically a within-subjects experimental study with follow-up interviews [42]. Assessment ratings were collected from recruited EM faculty participants, who were board-certified physicians in Canada or the USA and routinely supervise residents (e.g., work at an academic health center or teaching site for residents). From January 3rd, 2023, until June 28th, 2023, we recruited EM faculty from specialty networks and social media to complete an online survey, where they watched three scripted, standardized video performances of a *female* resident, each depicting a different clinical encounter, performing in the clinical workplace. These videos were developed by an EM and simulation fellowship trained faculty member (KN) and reviewed by an assessment expert for consistency and alignment to commonly assessed EM EPAs (SSS). Actors were used in all videos and followed a standardized script throughout filming (See Table 1 for details). After viewing each clip, faculty answered a consistent series of questions about whether they observed interdependence, and if they did, what kind: supportive or collaborative [8] (See Appendix). They also responded with open-text, narrative explanations after each smaller clip, entrustable professional activity (EPA) assessment, and Milestone rating in order to justify their judgements. At the beginning of each of the three videos faculty provided an EPA rating(s) for all EPAs that were applicable to the clinical encounter and were related to interdependence (akin to what would happen if a resident asked a faculty to evaluate them for a specific patient in the clinical workplace). After viewing all three videos, faculty also provided Milestone ratings for those that were applicable. This was done to reflect the understanding that milestones are generally assessed using data from more than one clinical encounter.

**Table 1:** Details of scripted scenarios and associated EPA assessments.


SCRIPTED SCENARIOS^a^	VIDEO DURATION	ACTORS PRESENT	EPA

Patient and Team Communication	3:35	Patient EM resident Nurse Pharmacist (On Voalte, voice only)	18: Communicate with other healthcare professionals about patient care

Performing Advanced Procedures: Intubation	7:18	Patient EMS (for handoff) Trauma surgeon EM attending EM resident Respiratory therapist Nurse	2: Lead the resuscitation of a critically ill or injured patient 11: Preform the diagnostic and therapeutic procedures of an emergency physician 12: Provide invasive and non-invasive airway management

Discharging a Patient	2:52	EM Resident EM Attending Nurse Social worker Medicine attending (on Voalte, voice only)	10: Develop and implement an appropriate disposition and aftercare plan


^a^Data from a female-presenting resident was collected from January to June 2023 using an online survey, and data from a male-presenting resident was collected from November 2023 to February 2024 using think aloud methods during a follow-up interview.

A second set of videos depicting a *male* resident performing the same three clinical cases was created. All other aspects of the videos were the same, including the other healthcare team members, clinical actions performed, content, and duration of the scenarios. We hired a professional videographer to match volume, tone, and pitch for both the female and male residents. About six months later, we followed up with a subset of faculty (18/63 participants) who agreed to be contacted for follow-up and asked them to complete a one-hour qualitative interview (rather than complete another online survey); these interviews occurred between November 28th, 2023, and February 24th, 2024. We selected an interview method for follow-up because we sought a more in-depth understanding about why faculty provided a particular rating, which we felt could be better elicited if prompted while completing the assessments. Using a different mode of data collection, combined with assessments over time, also may have reduced the extent to which gender was consciously considered by the participants. Interviews were conducted via zoom and involved a researcher taking the faculty through the same survey questions (see Appendix A) they completed previously, except they did this for a male-presenting resident. As part of the follow-up interviews, faculty were also asked questions about how they viewed interdependence for specific relationships. For example, the researchers shared examples where survey data collected from the larger group of faculty was divergent and asked questions such as: Could there be interdependence with patients here? Is this resident updating their supervisor an example of interdependence? Data from the online survey (female resident) were linked to the data collected during the qualitative interview (male resident) and quantitative analyses involved comparing EPA and Milestone ratings across the two video sets for each participant and were analyzed using the Wilcoxon signed-rank test. Transcribed data from narrative justifications of interdependence ratings (i.e., written for females and spoken for males), were analyzed iteratively using thematic analysis [43]. The analysis was sensitized by existing literature on gender bias (e.g., leadership expectations) in written assessments and narrative feedback [3,4,7,25]. Three members of the team (AE, SSS, and LL) separately reviewed the qualitative data, first familiarizing with the data through multiple readings, then generating codes for semantic meanings and latent meanings associated with our sensitizing literature. We compared results for consistency, and the confirmed codes were applied by AE to the entire dataset. Further group meetings considered connections and tensions evident in the completed coding and identified recurring themes. AE returned to the coded data to organize according to these themes, with iterative cycles of thematic coding and group discussion among all researchers to refine, elaborate and confirm the relevance of main themes.

Our work is shaped by our orientations to the research question, our disciplinary expertise, and our subject positions. SSS is a female measurement scientist who has a commitment to sophisticated measurement research that weaves together quantitative and qualitative methods to grapple with complex measurement problems. She also has experience researching gender equity issues during training and supervising female residents in their research activities. LL is a female rhetorician and qualitative methodologist whose work focuses on understanding the nuanced roles of language in clinical training and healthcare teamwork. She has a commitment to equity in her mentoring activities, and a longstanding awareness of the power of language to shape attitudes and actions. AE is a female medical student with an interest in medical education research and gender equity in training. We explicitly shared our experiences in group discussions as we coded and thematically analyzed the data, both in terms of moments of resonance with the findings and in terms of discrepancies, the latter being particularly important to refining our interpretations and understanding the questions that remain from this work.

## Results

A total of 18 faculty completed both the online survey and interview: ten females, and eight males. Eight faculty were from Canada and ten from the United States. Faculty ranged from 0 to over 31 years of practice (see Table 2).

**Table 2:** Descriptive Information of Survey Participants.


CHARACTERISTIC	EMERGENCY MEDICINE

**Gender, no. (%)**	

Female	10 (56%)

Male	8 (44%)

**Region in North America, no. (%)**	

Canada	8 (56%)

United States	10 (44%)

**Years in Practice, no. (%)**	

0–10	10 (56%)

11–20	4 (22%)

21–30	1 (5%)

31+	3 (17%)


Across the five EPA and two Milestone assessments, there were no statistically significant gender-based differences, except for one: the EPA assessment related to leading a resuscitation (z = –3.09, p = 0.002), where the male resident received lower entrustment ratings than the female resident (see Table 3). This exception may reflect heightened expectations for male residents in high-stakes leadership roles; a pattern explored further in our qualitative analysis.

**Table 3:** Faculty Rating Distributions of EPAs and Milestones for Female- and Male-Presenting Residents.^a^


ASSESSMENT TYPE	GENDER-PRESENTATION	1	2	3	4	5	*P* VALUE^b^

**EPA** ^c^							

Case 1 EPA: Communication	Female	0	0	5	5	8	.77

	Male	0	1	2	7	8	

Case 2 EPA: Procedures	Female	0	4	10	3	1	.93

	Male	0	4	9	5	0	

Case 2 EPA: Resuscitation	Female	0	0	7	9	2	.002**

	Male	0	8	8	2	0	

Case 2 EPA: Airway Management	Female	0	9	8	1	0	.74

	Male	0	8	9	1	0	

Case 3 EPA: Disposition Plan	Female	0	2	2	6	8	.56

	Male	0	1	3	4	10	

**Milestone**							

Systems-Based Practice 3: System Navigation for Patient Centered Care^d^	Female	1	2	11	3	1	.21

	Male	0	5	12	1	0	

Interprofessional and Communication Skills 2: Interprofessional and Team Communication^e^	Female	0	3	11	3	1	.56

	Male	0	1	13	3	1	


Abbreviations: EPA indicates Entrustable Professional Activity. ^a^Data from a female-presenting resident was collected from January to June 2023 using an online survey, and data from a male-presenting resident was collected from November 2023 to February 2024 using think aloud methods during a follow-up interview. ^b^From Wilcoxon signed-rank test; *P* is significant at .05. ^c^For EPA ratings, the response choices and anchors were as follows: 1 = I had to do it (Requires constant direct supervision and myself or others’ hands-on action for completion); 2 = I helped a lot (Requires considerable direct supervision and myself or others’ guidance for completion); 3 = I helped a little (Requires minimal direct supervision or guidance from myself or others for completion); 4 = I needed to be there but did not help (Requires indirect supervision and no guidance by myself or others); 5 = I didn’t need to be there at all (Does not require any supervision or guidance by myself or others). ^d^For the systems-based practice Milestone rating, across all three cases, the response choices and anchors were as follows: 1 = Level 1: Demonstrates knowledge of care coordination. Identifies key elements for safe and effective transitions of care and hand-offs. Demonstrates knowledge of population and community health needs and disparities; 2 = Level 2: In routine clinical situations, effectively coordinates patient care integrating the roles of interprofessional teams. In routine clinical situations, enables safe and effective transitions of care/hand-offs. Identifies specific population and community health needs and inequities for their local population; 3 = Level 3: In complex clinical situations, effectively coordinates patient care by integrating the roles of the interprofessional teams. In complex clinical situations, enables safe and effective transitions of care/hand-offs. Effectively uses local resources to meet the needs of a patient population and community; 4 = Level 4: Serves as a role model, effectively coordinates patient-centered care among different disciplines and specialties. Serves as a role model, advocates for safe and effective transitions of care/hand-offs within and across health care delivery systems, including outpatient settings. Participates in changing and adapting practice to provide for the needs of specific populations; 5 = Level 5: Analyzes the process of care coordination and leads in the design and implementation of improvements. Improves quality of transitions of care within and across health care delivery systems to optimize patient outcomes. Leads innovations and advocates for populations and communities with health care inequities. ^e^For the interprofessional and communication skills Milestone rating, across all three cases, the response choices and anchors were as follows: 1 = Level 1: Respectfully requests a consultation. Uses language that reflects the values all members of the health care team. Receives feedback in a respectful manner; 2 = Level 2: Clearly and concisely requests a consultation or other resources for patient care. Communicates information effectively with all health care team members; 3 = Level 3: Integrates recommendations made by various members of the health care team to optimize patient care. Engages in active listening to adapt to the communication styles of the team. Communicates concerns and provides feedback to peers and learners; 4 = Level 4: Acts as a role model for flexible communication strategies, i.e., those strategies that value input from all health care team members and that resolve conflict when needed. Uses effective communication to lead or manage health care teams. Communicates feedback and constructive criticism to superiors; 5 = Level 5: Acts as a role model for communication skills necessary to lead or manage health care teams. In complex situations, facilitates regular health care team-based feedback.

Qualitative analysis of narrative justifications identified differences in how faculty interpreted the same clinical performance for female and male residents. These differences were particularly evident when faculty talked about two features of interdependent performances: leadership and help-seeking behaviors. Below we illustrate recurring, gendered patterns using representative excerpts from the online survey and interview data. Faculty quotes are attributed using participant study identification number and gender (P#-F/M).

### Playing Second Fiddle: Leader versus Collaborator

#### Same Behaviour, Different Interpretations

Faculty described leadership behaviors differently for the same clinical performance depending on whether the resident was female or male. In the resuscitation video that was designed to demonstrate collaborative interdependence between an EM and a trauma resident, faculty consistently correctly identified the performance as collaborative interdependence. However, faculty justified and characterized the collaboration differently according to residents’ gender. For example, Participants 12, 38 and 13 described the male performance in terms of leadership: “he was leading it” (P12-F); “the trauma resident was doing the actual assessment, while the EM resident was leading it” (P38-M); or “The [EM] resident had more expertise in the area, and was providing more support to the trauma resident” (P13-F). In contrast, the same faculty assessing the same video described the female performance without referring to leadership. Faculty tended to characterize the performance as collaborative: “Collaborative initially…became supportive when resident required some guidance” (P12-F); “The EM resident and the trauma resident worked collaboratively to care for the patient” (P38-M); or there was “collaboration between the trauma resident and EM resident” (P13-F). Thus, while faculty were able to correctly identify collaborative interdependence for both male and female residents, they characterized collaboration differently by gender. This finding of contrasting language for male and female collaborative performances recurred across all 18 faculty.

#### Higher Expectations of Male Leadership

Although the male resident was more likely to be explicitly described as a leader, he was also criticized by faculty for perceived lack of leadership. In a clip depicting supportive interdependence where the resident, attending, a respiratory therapist, and nurse worked together to intubate a patient, one faculty said:

“The staff had to kind of say a few things that maybe he should have been able to do, and voice himself from the beginning. So, whether or not he knew the things that the staff was telling him, he didn’t voice them, which when you’re the leader of a team, you should be voicing those things from the beginning” (P63-F).

Here, the faculty acknowledges the resident may have lacked knowledge, and thus required support, but then critiqued the male resident’s failure to assume a more active, and vocal leadership role. Other faculty voiced similar concerns when commenting on that clip: “he didn’t you know, sort of do it like Superman all by himself” (P41-M) and used harsher language such as, “the resident was almost like the puppet and the staff was telling them exactly what to do” (P23-F). In a different clip, where the EM resident and the healthcare team were listening to a paramedic provide an overview of the patient’s condition, one faculty expressed their “disappointment that the EM resident stood there and didn’t do anything while he was listening” (P33-M).

Interestingly, in the same video, faculty did not make this observation of leadership absence for the female resident. Instead, they noted that the “consultant has to provide a lot of guidance” (P63-F) or “it looks like supportive [interdependence] because the attending provided minor corrections/supervision to the resident” (P41-M). Here, the use of more neutral language to describe the attending involvement does not imply a criticism of the female resident’s lack of leadership.

The key distinction between these quotes was the level of critique: the female resident’s need for guidance or support is described more matter-of-factly, while the male resident’s reliance on the consultant’s guidance suggested concerns about his ability to lead effectively. The discrepancy in these comments suggested higher expectations of leadership for male residents, even in situations where the male resident was being supported or guided. By contrast, female residents were not held to the same standard of leadership. Such patterns may also reflect different thresholds for what is considered ‘acceptable’ leadership based on gender—where male residents are scrutinized for not leading decisively, while female residents are not expected to lead in the same way. In fact, across all 18 faculty for all video clips, there was only one instance where a faculty explicitly used the word ‘leader’ when describing the female resident: “resident interacted with the paramedic and the trauma resident appropriately in a way that allows each team member to perform their role while she acted as leader.” (P12-F).

### Damsel in Distress: Help-Seeking versus Expertise-Seeking

#### Knowing When to Ask or Needing to Ask?

The other recurring pattern related to how faculty described female and male residents’ help-seeking behaviors. In a clip where a nurse notified the resident of a potential contraindication of a medication due to a patient’s condition, the male resident’s help-seeking was often perceived as demonstrating awareness of their limitations and taking initiative to seek help when necessary. For example, within the following justification, the faculty interpreted the male resident as actively acknowledging his limitations and seeking the pharmacist’s help appropriately: “the resident listened to the concerns of the nursing staff and then appropriately, when he felt it was beyond his expertise, reached out to the pharmacist” (P30-F). In contrast, the same faculty wrote that the female resident “required support from pharmacist to discuss side effects specific to medications that the resident didn’t know about” (P30-F). Direct, unhedged language like “required support” and “didn’t know about” characterizes female help-seeking as a result of lack of knowledge, while the male who “appropriately, when he felt it was beyond his expertise, reached out” is viewed more as proper judgement.

Another faculty assessing this clip stated that the male resident “recognizes that he doesn’t know and then he really realizes that he needs the pharmacist to address it” (P24-F), and for the female resident wrote, “the nurse had questions and concerns about the order, which she discussed with the resident who recognized the need to discuss with pharmacist” (P24-F). The first comment described the male resident’s agency and proactive role in recognizing their learning gaps and seeking help, while the second comment positioned the female resident as more passive and reactive, her recognition was merely a response to the nurse’s probing.

#### Help-Seeking as a Sign of Competence or Inexperience?

Moreover, although this video was designed to depict both supportive and collaborative interdependence, faculty interpreted the female resident’s actions as “supportive—inexperienced and lacking knowledge” (P9-M), while the male was viewed more precautionary, in that “they just wanted to kind of double check, and it didn’t seem quite dependent” (P9-M).

In instances where both the female and male residents sought the pharmacist’s input, the male resident’s behavior was described as “a hundred percent appropriate” (P37-F), reinforcing that his help-seeking was acceptable. Conversely, the female resident’s interaction with the pharmacist was described as a need for “help with information outside her area of expertise” (P37-F), which does not positively justify or explicitly affirm the appropriateness of her help-seeking.

#### Does Gender Influence Scope of Practice Expectations?

Regarding help-seeking, faculty demonstrated gendered expectations surrounding what is within EM scope of practice. For example, in the video described above where the resident consults with the pharmacist, one faculty claimed, “it was something that wasn’t within his main scope” (P12-F). In another video of a resident communicating with a social worker about appropriate disposition plans, a faculty noted, “I don’t think it’s within the scope of the resident’s practice to know what shelters or isolation shelters, etcetera, and that is where the social worker can come into play” (P38-M). Similarly, another faculty said, “this isn’t necessarily like he lacks something within his scope of practice” (P24-F). This language framed the male resident’s request for assistance as a matter of professional boundaries rather than a lack of knowledge, reinforcing a perception of the male resident’s competence. However, the female resident’s similar actions were viewed as reliance on external expertise due to a deficit in her own scope of practice. Comments such as “social work is using their expertise to help the resident understand the options for safe disposition” (P38-M), and “required social worker’s expertise and ability to contact shelters to craft a safe dispo plan” (P24-F) suggested that the expected “scope of practice” for the female and male residents differed despite identical training levels.

#### Assuming Expertise versus Questioning Expertise

Faculty perceptions of expertise also varied by resident gender. When assessing the male resident’s help-seeking behavior, there were several instances where faculty hesitated to attribute it to a lack of expertise. In the first video for example, when the resident sought the pharmacist’s help, faculty said “I don’t think that it was a lack of expertise” (P38-M), and further clarified that “it’s not that he lacks required expertise” (P63-F), “I don’t know if expertise is the right word, but just didn’t consider it maybe” (P9-M). Similarly, in another video, faculty reflected on the social worker’s guidance of the male resident and stated, “it was not necessarily an issue of expertise so much as just communicating a change in the situation” (P12-F). One faculty explicitly struggled with the term “lack of expertise” when describing a male resident and stated that the resident was “quite knowledgeable” (P30-F), thus softening any potential criticism. By contrast, descriptions of female resident’s gaps were more direct and specific, often noting a “lack of experience in decision-making” (P9-M) or “lack[ing] sufficient knowledge for important safety details” (P29-F), and finally “required support from pharmacist to discuss side effects specific to medications that the resident did not know about” (P30-F). This choice of language downplayed the male resident’s help-seeking and knowledge gaps, rephrasing it as circumstantial, and explicitly identified the female resident’s issues of inexperience or insufficient knowledge.

## Discussion

This study explored how gender influences the assessment of interdependence within the team-based environment of EM. When looking at the quantitative scores, we found no statistically significant differences based on gender for Milestone ratings or EPA ratings, except in the case of resuscitation. When looking beyond the scores, we identified disparities in terms of how faculty characterize and value interdependent behaviours that were influenced by resident’s gender, more specifically their leadership and help-seeking. These findings suggest that bias may not always manifest in scoring, but rather, in the interpretive layer of assessment—how competence is seen, framed, and valued.

### Surface Equality or Subtle Inequity? What the Numbers Miss

Our quantitative results align with previous research reporting no significant differences in milestone or simulation-based scores between male and female EM residents under controlled conditions [18,44,45]. This discrepancy between scores and narrative interpretation may lead some readers to conclude that gender bias is absent from assessment. However, this interpretation overlooks important contextual factors. Our study design for example, involved scripted and directly observed scenarios, which reduces the influence of unconscious bias. Additionally, the timing of assessments is another factor to consider. Since assessments in simulation-based environments are made directly after observation, this finding suggests that immediate, performance-focused assessments may help reduce implicit bias. Longitudinal studies examining gender bias in resident assessments demonstrate that, over time, male residents achieve higher milestone scores and at a faster rate than female residents [19,20]. These differences in trends and widening of gender gaps may be attributable to the cumulative effect of recurrent biases and obstacles that are more pronounced at higher levels of training [20].

The structure of our study, combined with limited statistical power, create conditions where overt score disparities may be less likely to surface. To further understand why bias can be present despite score parity, we turn to Correll and colleagues’ (2020) “view and value” model [46]. This framework suggests that evaluators may *observe* similar behaviours across individuals (view) but *assign* different meanings or importance (value) to those behaviours based on social norms and gendered expectations. In our study, this distinction between what is *scored* and how performance is *interpreted* was made visible through qualitative analysis. Our results highlight a key insight: the absence of score differences do not equate with the absence of bias. Thus, the real insight of this study lies not in the numerical data alone, but in how narrative feedback reveals underlying gendered assumptions that shape the assessment of interdependent behaviours.

### Damned if You Do, Critiqued if You Don’t: The Double-Edged Sword of Leadership

Our qualitative analysis identified gendered patterns in the narrative justifications, particularly around leadership and help-seeking, which highlights the intersections between gender bias and interdependence. In terms of leadership, faculty often portrayed the male resident as a leader, explicitly identifying their role in directing and guiding teams, while the female resident performing the same tasks was described as collaborative or supportive. This echoes findings from other studies noting that female resident comments emphasize “communal” traits: nurturing, kind, dependent, while male residents receive comments noting their “agentic” traits: strong, logical, independent [47,48,49]. These trends reflect stereotypical male assumptions about the ideal leader [49,50]. Such biases pose challenges for female residents in leadership roles, especially in male-dominant specialties like EM that have unspoken expectations of “masculine” traits [49,51].

This stereotype of a masculine leader can disadvantage both male and female residents. Paradoxically, male residents were not only more likely to be framed as leaders, but also more likely to be criticized when leadership was perceived to be lacking. Female residents by contrast were rarely recognized as leaders and rarely penalized for not leading despite performing the same clinical encounter. Such discrepancies may stem from underlying societal expectations, which contribute complexity to the assessment impacts for both male and female residents. For males, a “double-edged sword” effect may happen, whereby they are expected to lead confidently, and the absence of such behavior is viewed critically [52]. For females, a “damned if you do, critiqued if you don’t” effect may emerge due to the association of leadership with “masculine” traits. Females with non-stereotypical presentations of leadership may not be recognized as leading while those who adapt their behaviors to reflect stereotypical leadership traits like direct and assertive actions may be met with negative evaluations —and reminders to remain “nice,” “polite,” and avoid being “bossy”—for displaying behavior deemed overly (un-femininely) agentic [31,45,46,49,50,51,52,53,54,55,56,57]. These patterns expose the limitations of traditional leadership archetypes, especially in male-dominated fields like EM, and point to the need for a broader, more inclusive conception of leadership. Thus, recognizing the interplay of gendered leadership assumptions, and redefining leadership in residency in ways that emphasize and reward interdependence, can help ensure that all residents are assessed equitably for leadership, regardless of gender [49,50].

### Needing Help vs Seeking Expertise? The Cost of Asking for Help

Our findings also highlight gendered nuances in faculty interpretations of residents’ help-seeking behaviors. The female resident was frequently described as “requiring support” or “needing help,” whereas the male resident was depicted as “recognizing their limitations” and “seeking expertise.” This language suggests an underlying stereotype rooted in benevolent sexism, which—though seemingly positive by promoting prosocial behaviors like helping—reinforces sexist norms that position females as a weaker sex [58]. This stereotype also influences the perception of competence in the workforce, where males may be seen as experts and competent by default, while females face assumptions of dependency despite similar performance and qualifications [59,60,61,62].

Assumptions affect how help-seeking behaviors are perceived. When male residents are presumed inherently competent, help-seeking may be framed as an initiative to supplement learning, whereas for female residents, it is framed as requiring support to fill a knowledge gap. These gendered expectations can influence perceptions of interdependence in a team environment, creating different approaches to “helping” residents based on gender. Studies on dependency-oriented help (e.g., providing solutions) versus autonomy-oriented help (e.g., equipping with tools to problem-solve independently) found that females, especially in traditionally “masculine” domains, are more likely to receive dependency-oriented help, which is associated with lower perceived competence [63,64]. Conversely, autonomy-oriented help was more often granted to males, who were seen as capable.

These differing perceptions have important implications for performance assessments within team-based clinical environments. Male residents may be seen as more autonomous and independent, while female residents may be seen as requiring ongoing support, even when exhibiting the same behaviours. This subtle language difference in assessments can penalize female residents for seeking help and discourage male residents from doing so, reinforcing a cycle that dissuades both genders from asking for guidance when needed [65,66]. This reluctance can be dangerous in a field that values collaboration, input, and guidance to reduce errors and improve patient outcomes. To counter these effects, assessments could benefit by focusing less on the individual and rewarding aspects such as cooperation and cohesion regardless of gender. Ultimately, the patterns we observed in our study suggest that even when behaviours are identical, the meanings ascribed to them may differ by gender, underscoring the need for caution when drawing conclusions from narrative assessments in interdependent clinical contexts.

### Leveraging Evaluation to Understand the Assessment Gap: Lessons Learned

Given our findings, what does it mean that we observed gender bias in qualitative assessments, but not necessarily in quantitative scores? To answer this question, firstly, we argue that the lack of score disparity may reflect the standardized and controlled design of our study as opposed to the absence of score bias. Furthermore, the disconnect between quantitative parity and qualitative bias challenges a common assumption in medical education: that narrative feedback complements and enriches numerical scores. In our study, we found that narrative comments, rather than offering neutral or clarifying insights, introduced bias at times that reflected gendered expectations that shaped the interpretation of competence, sometimes undermining the equity suggested by score parity.

Returning to Correll and colleagues’ “view and value” model (2020), this disconnect becomes clearer [46]. The extent to which faculty may *view* similar performances by male and female residents, can differ from how they *value* that trait or behaviour depending on gender norms. This framing helps explain why identical behaviours such as “communicating with the health care professionals” or “leading the resuscitation of a critically ill patient,” resulted in similar EPA scores. However, when elaborating on the resident performance and their interdependence, assessors may have *valued* certain traits such as leadership, collaboration, and help-seeking differently, resulting in different gendered interpretations. Therefore, it is not that males and females are being evaluated differently as residents; rather, faculty expect residents to enact their job as gendered people, and consequently police behaviors that fall outside those expectations [46].

Recent studies suggest that EPA scores alone may fail to capture important contextual information needed to accurately assess a resident’s competence and these studies are often used to foreground the value of narrative comments [67,68,69]. These findings caution against the uncritical acceptance of narrative feedback, particularly in high-stakes decisions. Rather than treating narratives as neutral additions to numeric data, we must recognize them as interpretive acts shaped by social norms—including those related to gender. We encourage educators and clinical competency committees to continue to evaluate (just as we have done with this study) and share their lessons learned about how the quantitative and qualitative assessment data they use can differently reflect gender bias, especially as it relates to residents’ performances as members of the clinical team [70,71,72]. As interdependence becomes an increasingly important marker of competence in collaborative, team-based specialties and their assessments of such, equity in how these behaviors are interpreted and assessed must become a priority.

### Limitations

While the small sample size of 18 faculty participants limits the generalizability of our findings, particularly in interpreting the absence of significant differences in EPA and Milestone scores, it is strengthened by our within-subjects design, in which each participant assessed both a male and a female resident. Additionally, by sampling across American and Canadian training contexts, we strengthen the likelihood that our insights are transferrable to other settings that share training characteristics with North America. With this in mind, the absence of scoring differences needs to be interpreted with caution, as the small sample may have limited our ability to detect score-based disparities, especially considering potential ceiling effects in EPA ratings. It is plausible that gendered perceptions were present but not captured numerically due to these constraints. Additionally, participants were self-selected, potentially resulting in a group that was more equity-aware or sensitized to issues of bias than the broader population of EM faculty. This awareness could have influenced the nature of the assessments, particularly in the Milestone and EPA ratings. Our study also spanned a six-month data collection period, which introduces the possibility of temporal changes in participants’ thinking, training experiences, or sensitivity to bias—factors that may have influenced their assessments between the survey and interview phases.

Using two different data collection methods, online surveys (written feedback for female residents), and semi-structured interviews (spoken feedback for male residents) introduces variability that somewhat complicates direct comparisons of male and female resident performances. This was, however, a deliberate methodological choice: in collecting data using different methods, we aimed to reduce gender priming, and facilitate candid, detailed reflections. Additionally, both sets of data were linked at the participant level, and themes were triangulated with care. Neither written nor interview data are necessarily better or worse, but they are importantly different: interview data, while potentially richer, were collected in a non-anonymous setting, while written data were collected anonymously. This distinction introduces potential biases: interview data may be influenced by the Hawthorne effect, as faculty knew a research member was recording their assessments. In contrast, the anonymity of written assessments might have allowed for more candid critiques, but possibly limited due to the additional effort involved in writing comments, which may have deterred faculty from providing detailed justifications. We also acknowledge that the Milestones and EPAs used were not explicitly designed to measure interdependence—a key construct in our study—but they offered a structured framework in the absence of established tools, which further emphasize the importance of our study. Our goal was not to validate these tools for interdependence, but rather to explore how existing assessment mechanisms may reflect gendered interpretations of interdependent behaviours.

Future studies that employ consistent data collection methods with larger samples of male and female faculty will help to tease apart the gendered patterns we identified from the methods of data collection and help determine whether gendered patterns exist within subsets of faculty.

## Conclusion

This study extends the research on gender bias by examining its influence on the characterization of interdependence in clinical performance assessments. Our findings show that although Milestone and EPA scores suggested marginal gender-based differences, the narrative justifications reflected clear gendered interpretations and expectations about how residents perform as team members. These insights emphasize the limitations of quantitative or qualitative metrics alone and highlight the importance of examining both quantitative and qualitative dimensions of assessment to identify and mitigate gender bias, In doing so, we can ensure that competency-based education in EM and other team-based clinical training environments provides fair and equitable assessments of performance, regardless of gender.

## Previous Presentations

Preliminary data was presented at the Centre for Education Research and Innovation, October 8, 2024, London, Ontario, Canada.

## Additional File

The additional file for this article can be found as follows:

10.5334/pme.1937.s1 Appendix A. EM Survey: Conceptualizing &Assessing Interdependent Performance.
