# Supplementary material for: Beyond the Scores: Gendered Interpretations of Emergency Medicine Resident Assessments of Interdependent Performances
Source: Perspect Med Educ. 2025 Aug 21;14(1):504–18. doi: 10.5334/pme.1937 (PMC12372684; doi:10.5334/pme.1937)
Supplement: Appendix A. — EM Survey: Conceptualizing &Assessing Interdependent Performance. [file pme-14-1-1937-s1.pdf]

EM Survey: Conceptualizing & Assessing Interdependent Performance

Start of Block: Default Question Block

Q1 Letter of Information for Participation in a Research Study
Study Title: Conceptualizing & Assessing Interdependent Performance in Collaborative Clinical Environments
Study PI: Lorelei Lingard (PI), PhD
Study co-PI: Stefanie Sebok-Syer, PhD

INTRODUCTION
You are being invited to participate in a research study. You are invited to participate in this study because you are a member of the clinical team in either Emergency Medicine or Pediatrics and you completed the previous survey and indicated interest in participating in a follow-up interview. This consent form provides you with information to help you make an informed choice. Please read this document carefully and ask any questions that may arise. Taking part in this study is voluntary. Deciding not to take part or deciding to leave the study later will not result in any penalty or affect current or future employment.

IS THERE A CONFLICT OF INTEREST?
There are no conflicts of interest to declare related to this study.

WHY IS THIS STUDY BEING DONE?
The purpose of this study to develop, pilot and validate a new conceptualization of trainees' interdependent performances in collaborative clinical settings. You are being asked to participate in the third phase of this work, in which we will conduct individual interviews to explore the suitability of data generated by our new conceptualization for use in decisions regarding trainee progression. Additionally, we are aiming to explore a variety of factors that might influence interdependence assessments.

HOW MANY PEOPLE WILL TAKE PART IN THIS STUDY?
It is anticipated that 25-30 individuals will be interviewed in this phase. This phase will take approximately 3 months to complete.

WHAT WILL HAPPEN DURING THIS STUDY?
You will be asked to schedule/attend an interview. During this interview, you will meet with a member of the research team. Each interview will be about 60 minutes in length and will take place via telephone or Zoom. Consent to be audio recorded will be requested prior to beginning the interview. Please be advised that by accepting to be interviewed, you are also accepting to

be audio recorded as it is mandatory. You will be given and asked to sign a consent form prior to commencing the interview
During the interview, you will be shown video clips and asked questions related to broad patterns in the results. Questions will focus on each type of interdependence, including key features and assessment-focused questions. Questions will also address some factors that may influence assessments of interdependence performance of trainees.

WHAT ARE THE RESPONSIBILITIES OF STUDY PARTICIPANTS?
If you choose to participate in this study, you will be expected to complete an interview with a trained researcher about a new conceptualization of assessing interdependent performances.

HOW LONG WILL PARTICIPANTS BE IN THE STUDY?
Your participation on this study will last for the hour in which you are interviewed for the study.

CAN PARTICIPANTS CHOOSE TO LEAVE THE STUDY?
You can choose to end your participation in this research (called withdrawal) at any time without having to provide a reason. If you choose to withdraw from the study, you are encouraged to contact the research team.
You may withdraw your permission to use information that was collected about you for this study at any time by letting the research team know.
If you decide to leave the study, you can ask that the information that was collected about you not be used for the study. Let the research team know if you choose this.

WHAT ARE THE RISKS OR HARMS OF PARTICIPATING IN THIS STUDY?
Risks of privacy breach.

WHAT ARE THE BENEFITS OF PARTICIPATING IN THIS STUDY?
Participants will receive a $50 Amazon gift-card. A research assistant will send the gift card over email.

HOW WILL PARTICIPANT INFORMATION BE KEPT CONFIDENTIAL?
The principal investigator will be serving as the data custodian. In addition, representatives of of the Western University Health Sciences Research Ethics Board may require access to your study-related documents to oversee the ethical conduct of this study.

Study data in the form of analyses will be stored on a memory stick with an encrypted password, as well as on a laptop in the locked campus office of the researchers.

The following identifiers will be collected: full name and email address.

OneDrive will be used as a secure storage site for electronic research materials and for sending data to Stanford.

Qualtrics will be used to collect survey data. Qualtrics' privacy policy can be found here:

https://www.qualtrics.com/privacy-statement/. The data controller is located in the United States of America. There are always risks when storing data on the internet, such as a privacy breach.

If the results of this study are published, your identity will remain confidential. It is expected that the information collected during this study will be used in analyses and will be published/presented to the scientific community at meetings and in journals.

All study data will be kept for 7 years after which it will be destroyed.

WHAT IS THE COST TO PARTICIPANTS?
Participation in this study will not involve any additional costs to you.

WHAT ARE THE RIGHTS OF PARTICIPANTS IN A RESEARCH STUDY?
You will be told, in a timely manner, about new information that may be relevant to your willingness to stay in this study. You have the right to be informed of the results of this study once the entire study is complete. Your rights to privacy are legally protected by federal and provincial laws that require safeguards to ensure that your privacy is respected. By consenting, you do not give up any of your legal rights against the researcher or involved institutions for compensation, nor does this form relieve the researcher or their agents of their legal and professional responsibilities.

WHOM DO PARTICIPANTS CONTACT FOR QUESTIONS?
If you have questions about taking part in this study, you can talk to the principle investigator.
That person is: Lorelei Lingard 519-661-2111 ext. 88999.
If you have any questions about your rights as a research participant or the conduct of the study you may contact the office of Human Research Ethics at 519.661.3036 or ethics@uwo.ca.
Sincerely,
Lorelei Lingard, PhD

I acknowledge that:
All my questions have been answered.
I understand the information within the informed consent form.
I have read, or someone has read to me, each page of this participant informed consent form.
I do not give up any of my legal rights by signing this consent form.
I agree to take part in this study.
Participation in the survey will be taken as your implied consent to participate.

Page Break

Q2 In which state or province is your practice located?

▼ Alabama (1) ... Saskatchewan (63)

Q3 How many years have you been working with residents?

____

Page Break

Q5 This is the EM SENIOR RESIDENT that you will be assessing throughout this survey:

Nurse Faculty Pharmacist Consult

Case 1 Please watch the entire video before proceeding.

Q6 CASE 1 EPA: COMMUNICATE WITH OTHER HEALTHCARE PROFESSIONALS ABOUT PATIENT CARE.
Which EPA rating would you give to this EM resident?

○ 1. I had to do it (Requires constant direct supervision and myself or others' hands-on action for completion). (1)

○ 2. I helped a lot (Requires considerable direct supervision and myself or others' guidance for completion). (9)

○ 3. I helped a little (Requires minimal direct supervision or guidance from myself or others for completion). (2)

○ 4. I needed to be there but did not help (Requires indirect supervision and no guidance by myself or others). (3)

○ 5. I didn't need to be there at all (Does not require any supervision or guidance by myself or others). (10)

Page Break

Q7 **1.1** Now you will be asked questions related to four specific clips from the previous video. Please review as needed to refresh your memory.

Q8 **1.1** Interdependence definition
Patterns of interactions between individuals that can afford or constrain one's performance and shape the practice of the broader healthcare team.

Did you observe interdependence between the EM RESIDENT and anyone else in this clip?

○ Yes (1)

○ No (2)

Page Break

Display This Question:

If 1.1 Interdependence definition Patterns of interactions between individuals that can afford or co... = No

Q9 **1.1** Why did you answer 'no' to the previous question?

____

Page Break

Display This Question:

If 1.1 Interdependence definition Patterns of interactions between individuals that can afford or co... = Yes

Q10 **1.1** Which interdependence did you observe?

○ Supportive Interdependence [Triggered by a resident's lack of expertise within their scope of practice.] (1)

○ Collaborative Interdependence [Triggered by a recognition that patient care requires contributions from other team members.] (2)

○ Both (3)

Page Break

Display This Question:

If 1.1 Interdependence definition Patterns of interactions between individuals that can afford or co... = Yes

And 1.1 Which interdependence did you observe? = Supportive Interdependence [Triggered by a resident's lack of expertise within their scope of practice.]

Or 1.1 Which interdependence did you observe? = Collaborative Interdependence [Triggered by a recognition that patient care requires contributions from other team members.]

Q11 **1.1** Please explain why you selected this type of interdependence.

____

Page Break

Display This Question:

If 1.1 Which interdependence did you observe? = Both

Q12 **1.1** Please explain why you selected 'both'.

____

Page Break

Q13 **1.2** Please review this clip as needed to refresh your memory.

Q14 **1.2** Interdependence definition
Patterns of interactions between individuals that can afford or constrain one's performance and shape the practice of the broader healthcare team.

Did you observe interdependence between the EM RESIDENT and anyone else in this clip?

- ○ Yes (1)
- ○ No (2)

Page Break

Display This Question:

If 1.2 Interdependence definition Patterns of interactions between individuals that can afford or co... = No

Q15 **1.2** Why did you answer 'no' to the previous question?

____

Page Break

Display This Question:

If 1.2 Interdependence definition Patterns of interactions between individuals that can afford or co... = Yes

Q16 **1.2** Which interdependence did you observe?

○ Supportive Interdependence [Triggered by a resident's lack of expertise within their scope of practice.] (1)

○ Collaborative Interdependence [Triggered by a recognition that patient care requires contributions from other team members.] (2)

○ Both (3)

Page Break

Display This Question:

If 1.2 Interdependence definition Patterns of interactions between individuals that can afford or co... = Yes

And 1.2 Which interdependence did you observe? = Supportive Interdependence [Triggered by a resident's lack of expertise within their scope of practice.]

Or 1.2 Which interdependence did you observe? = Collaborative Interdependence [Triggered by a recognition that patient care requires contributions from other team members.]

Q17 **1.2** Please explain why you selected this type of interdependence.

____

Page Break

Display This Question:

If 1.2 Which interdependence did you observe? = Both

Q18 **1.2** Please explain why you selected 'both'.

____

Page Break

Q19 **1.3** Please review this clip as needed to refresh your memory.

Q20 **1.3** Interdependence definition
Patterns of interactions between individuals that can afford or constrain one's performance and shape the practice of the broader healthcare team.

Did you observe interdependence between the EM RESIDENT and anyone else in this clip?

- ○ Yes (1)
- ○ No (2)

Page Break

Display This Question:

If 1.3 Interdependence definition Patterns of interactions between individuals that can afford or co... = No

Q21 **1.3** Why did you answer 'no' to the previous question?

____

Page Break

Display This Question:

If 1.3 Interdependence definition Patterns of interactions between individuals that can afford or co... = Yes

Q22 **1.3** Which interdependence did you observe?

○ Supportive Interdependence [Triggered by a resident's lack of expertise within their scope of practice.] (1)

○ Collaborative Interdependence [Triggered by a recognition that patient care requires contributions from other team members.] (2)

○ Both (3)

Page Break

Display This Question:

If 1.3 Which interdependence did you observe? = Both

Q23 **1.3** Please explain why you selected 'both'.

____

Page Break

Display This Question:

If 1.3 Interdependence definition Patterns of interactions between individuals that can afford or co... = Yes

And 1.3 Which interdependence did you observe? = Supportive Interdependence [Triggered by a resident's lack of expertise within their scope of practice.]

Or 1.3 Which interdependence did you observe? = Collaborative Interdependence [Triggered by a recognition that patient care requires contributions from other team members.]

Q24 **1.3** Please explain why you selected this type of interdependence.

____

Page Break

Q25 **1.4** Please review this clip as needed to refresh your memory.

Q26 **1.4** Interdependence definition
Patterns of interactions between individuals that can afford or constrain one's performance and shape the practice of the broader healthcare team.

Did you observe interdependence between the EM RESIDENT and anyone else in this clip?

- ○ Yes (1)
- ○ No (2)

Page Break

Display This Question:

If 1.4 Interdependence definition Patterns of interactions between individuals that can afford or co... = No

Q27 **1.4** Why did you answer 'no' to the previous question?

____

Page Break

Display This Question:

If 1.4 Interdependence definition Patterns of interactions between individuals that can afford or co... = Yes

Q28 **1.4** Which interdependence did you observe?

- ◯ Supportive Interdependence [Triggered by a resident's lack of expertise within their scope of practice.] (1)
- ◯ Collaborative Interdependence [Triggered by a recognition that patient care requires contributions from other team members.] (2)
- ◯ Both (3)

Page Break

Display This Question:

If 1.4 Which interdependence did you observe? = Both

Q29 **1.4** Please explain why you selected 'both'.

____

Page Break

Display This Question:

If 1.4 Interdependence definition Patterns of interactions between individuals that can afford or co... = Yes

And 1.4 Which interdependence did you observe? = Supportive Interdependence [Triggered by a resident's lack of expertise within their scope of practice.]

Or 1.4 Which interdependence did you observe? = Collaborative Interdependence [Triggered by a recognition that patient care requires contributions from other team members.]

Q30 **1.4** Please explain why you selected this type of interdependence.

____

Page Break

Q31 Case 1: Overall, was the degree of interdependence appropriate based on the whole scenario?

○ Yes (1)

○ No (2)

Q32 Please explain why or why not.

____

Page Break

Q33 **Case 2** Please watch the entire video before proceeding.

Resident	Trauma		
Resident	Faculty	Nurse	Respiratory Therapist

Q34 Please provide ratings for the three following EPAs:
Lead the resuscitation of a critically ill or injured patient.
Perform the diagnostic and therapeutic procedures of an emergency physician.
Provide invasive and non-invasive airway management.

CASE 2 EPA: LEAD THE RESUSCITATION OF A CRITICALLY ILL OR INJURED PATIENT.
Which EPA rating would you give to this EM resident?

○ 1. I had to do it (Requires constant direct supervision and myself or others' hands-on action for completion). (1)

○ 2. I helped a lot (Requires considerable direct supervision and myself or others' guidance for completion). (7)

○ 3. I helped a little (Requires minimal direct supervision or guidance from myself or others for completion). (2)

○ 4. I needed to be there but did not help (Requires indirect supervision and no guidance by myself or others). (3)

○ 5. I didn't need to be there at all (Does not require any supervision or guidance by myself or others). (8)

Q35 CASE 2 EPA: PERFORM THE DIAGNOSTIC AND THERAPEUTIC PROCEDURES OF AN EMERGENCY PHYSICIAN.
Which EPA rating would you give to this EM resident?

- ○ 1. I had to do it (Requires constant direct supervision and myself or others' hands-on action for completion). (1)
- ○ 2. I helped a lot (Requires considerable direct supervision and myself or others' guidance for completion). (7)
- ○ 3. I helped a little (Requires minimal direct supervision or guidance from myself or others for completion). (2)
- ○ 4. I needed to be there but did not help (Requires indirect supervision and no guidance by myself or others). (3)
- ○ 5. I didn't need to be there at all (Does not require any supervision or guidance by myself or others). (8)

Q36 CASE 2 EPA: PROVIDE INVASIVE AND NON-INVASIVE AIRWAY MANAGEMENT.
Which EPA rating would you give to this EM resident?

- ○ 1. I had to do it (Requires constant direct supervision and myself or others' hands-on action for completion). (1)
- ○ 2. I helped a lot (Requires considerable direct supervision and myself or others' guidance for completion). (7)
- ○ 3. I helped a little (Requires minimal direct supervision or guidance from myself or others for completion). (2)
- ○ 4. I needed to be there but did not help (Requires indirect supervision and no guidance by myself or others). (3)
- ○ 5. I didn't need to be there at all (Does not require any supervision or guidance by myself or others). (8)

Page Break

Q37 **2.1** Now you will be asked questions related to four specific clips from the previous video. Please review as needed to refresh your memory.

Q38 **2.1** Interdependence definition
Patterns of interactions between individuals that can afford or constrain one's performance and shape the practice of the broader healthcare team.

Did you observe interdependence between the EM RESIDENT and anyone else in this clip?

- ◯ Yes (1)
- ◯ No (2)

Page Break

Display This Question:

If 2.1 Interdependence definition Patterns of interactions between individuals that can afford or co... = No

Q39 **2.1** Why did you answer 'no' to the previous question?

____

Page Break

Display This Question:

If 2.1 Interdependence definition Patterns of interactions between individuals that can afford or co... = Yes

Q40 **2.1** Which interdependence did you observe?

- ◯ Supportive Interdependence [Triggered by a resident's lack of expertise within their scope of practice.] (1)
- ◯ Collaborative Interdependence [Triggered by a recognition that patient care requires contributions from other team members.] (2)
- ◯ Both (3)

Page Break

Display This Question:

If 2.1 Interdependence definition Patterns of interactions between individuals that can afford or co... = Yes

And 2.1 Which interdependence did you observe? = Supportive Interdependence [Triggered by a resident's lack of expertise within their scope of practice.]

Or 2.1 Which interdependence did you observe? = Collaborative Interdependence [Triggered by a recognition that patient care requires contributions from other team members.]

Q41 **2.1** Please explain why you selected this type of interdependence.

____

Page Break

Display This Question:

If 2.1 Which interdependence did you observe? = Both

Q42 **2.1** Please explain why you selected 'both'.

____

Page Break

Q43 **2.2** Please review this clip as needed to refresh your memory.

Q44 **2.2** Interdependence definition
Patterns of interactions between individuals that can afford or constrain one's performance and shape the practice of the broader healthcare team.

Did you observe interdependence between the EM RESIDENT and anyone else in this clip?

○ Yes (1)

○ No (2)

Page Break

Display This Question:

If 2.2 Interdependence definition Patterns of interactions between individuals that can afford or co... = No

Q45 **2.2** Why did you answer 'no' to the previous question?

____

Page Break

Display This Question:

If 2.2 Interdependence definition Patterns of interactions between individuals that can afford or co... = Yes

Q46 **2.2** Which interdependence did you observe?

◯ Supportive Interdependence [Triggered by a resident's lack of expertise within their scope of practice.] (1)

◯ Collaborative Interdependence [Triggered by a recognition that patient care requires contributions from other team members.] (2)

◯ Both (3)

Page Break

Display This Question:

If 2.2 Interdependence definition Patterns of interactions between individuals that can afford or co... = Yes

And 2.2 Which interdependence did you observe? = Supportive Interdependence [Triggered by a resident's lack of expertise within their scope of practice.]

Or 2.2 Which interdependence did you observe? = Collaborative Interdependence [Triggered by a recognition that patient care requires contributions from other team members.]

Q47 **2.2** Please explain why you selected this type of interdependence.

____

Page Break

Display This Question:

If 2.2 Which interdependence did you observe? = Both

Q48 **2.2** Please explain why you selected 'both'.

____

Page Break

Q49 **2.4** Please review this clip as needed to refresh your memory.

Q50 **2.4** Interdependence definition
Patterns of interactions between individuals that can afford or constrain one's performance and shape the practice of the broader healthcare team.

Did you observe interdependence between the EM RESIDENT and anyone else in this clip?

- Yes (1)
- No (2)

Page Break

Display This Question:

If 2.4 Interdependence definition Patterns of interactions between individuals that can afford or co... = No

Q51 **2.4** Why did you answer 'no' to the previous question?

____

Page Break

Display This Question:

If 2.4 Interdependence definition Patterns of interactions between individuals that can afford or co... = Yes

Q52 **2.4** Which interdependence did you observe?

○ Supportive Interdependence [Triggered by a resident's lack of expertise within their scope of practice.] (1)

○ Collaborative Interdependence [Triggered by a recognition that patient care requires contributions from other team members.] (2)

○ Both (3)

Page Break

Display This Question:

If 2.4 Interdependence definition Patterns of interactions between individuals that can afford or co... = Yes

And 2.4 Which interdependence did you observe? = Supportive Interdependence [Triggered by a resident's lack of expertise within their scope of practice.]

Or 2.4 Which interdependence did you observe? = Collaborative Interdependence [Triggered by a recognition that patient care requires contributions from other team members.]

Q53 **2.4** Please explain why you selected this type of interdependence.

____

Page Break

Display This Question:

If 2.4 Which interdependence did you observe? = Both

Q54 **2.4** Please explain why you selected 'both'.

____

Page Break

Q55 **2.5** Please review this clip as needed to refresh your memory.

Q56 **2.5** Interdependence definition
Patterns of interactions between individuals that can afford or constrain one's performance and shape the practice of the broader healthcare team.

Did you observe interdependence between the EM RESIDENT and anyone else in this clip?

- ○ Yes (1)
- ○ No (2)

Page Break

Display This Question:

If 2.5 Interdependence definition Patterns of interactions between individuals that can afford or co... = No

Q57 **2.5** Why did you answer 'no' to the previous question?

____

Page Break

Display This Question:

If 2.5 Interdependence definition Patterns of interactions between individuals that can afford or co... = Yes

Q58 **2.5** Which interdependence did you observe?

- ◯ Supportive Interdependence [Triggered by a resident's lack of expertise within their scope of practice.] (1)
- ◯ Collaborative Interdependence [Triggered by a recognition that patient care requires contributions from other team members.] (2)
- ◯ Both (3)

Page Break

Display This Question:

If 2.5 Which interdependence did you observe? = Both

Q59 **2.5** Please explain why you selected 'both'.

____

Page Break

Display This Question:

If 2.5 Interdependence definition Patterns of interactions between individuals that can afford or co... = Yes

And 2.5 Which interdependence did you observe? = Supportive Interdependence [Triggered by a resident's lack of expertise within their scope of practice.]

Or 2.5 Which interdependence did you observe? = Collaborative Interdependence [Triggered by a recognition that patient care requires contributions from other team members.]

Q60 **2.5** Please explain why you selected this type of interdependence.

____

Page Break

Q61 Case 2: Overall, was the degree of interdependence appropriated based on the whole scenario?

- Yes (1)
- No (2)

Q62 Please explain why or why not.

____

Page Break

Q63 **Case 3** Please watch the entire video before proceeding.

Resident	Faculty	Nurse	Social Worker

Q64 CASE 3 EPA: DEVELOP AND IMPLEMENT AN APPROPRIATE DISPOSITION AND AFTERCARE PLAN.
Which EPA rating would you give to this EM resident?

- ○ 1. I had to do it (Requires constant direct supervision and myself or others' hands-on action for completion). (1)
- ○ 2. I helped a lot (Requires considerable direct supervision and myself or others' guidance for completion). (6)
- ○ 3. I helped a little (Requires minimal direct supervision or guidance from myself or others for completion). (2)
- ○ 4. I needed to be there but did not help (Requires indirect supervision and no guidance by myself or others). (3)
- ○ 5. I didn't need to be there at all (Does not require any supervision or guidance by myself or others). (7)

Page Break

Q65 **3.2** Now you will be asked questions related to three specific clips from the previous video. Please review as needed to refresh your memory.

Q66 **3.2** <u>Interdependence definition</u>
Patterns of interactions between individuals that can afford or constrain one's performance and shape the practice of the broader healthcare team.

Did you observe interdependence between the EM RESIDENT and anyone else in this clip?

- ○ Yes (1)
- ○ No (2)

Page Break

Display This Question:

If 3.2 Interdependence definition Patterns of interactions between individuals that can afford or co... = No

Q67 **3.2** Why did you answer 'no' to the previous question?

____

Page Break

Display This Question:

If 3.2 Interdependence definition Patterns of interactions between individuals that can afford or co... = Yes

Q68 **3.2** Which interdependence did you observe?

○ Supportive Interdependence [Triggered by a resident's lack of expertise within their scope of practice.] (1)

○ Collaborative Interdependence [Triggered by a recognition that patient care requires contributions from other team members.] (2)

○ Both (3)

Page Break

Display This Question:

If 3.2 Interdependence definition Patterns of interactions between individuals that can afford or co... = Yes

And 3.2 Which interdependence did you observe? = Supportive Interdependence [Triggered by a resident's lack of expertise within their scope of practice.]

Or 3.2 Which interdependence did you observe? = Collaborative Interdependence [Triggered by a recognition that patient care requires contributions from other team members.]

Q69 **3.2** Please explain why you selected this type of interdependence.

____

Page Break

Display This Question:

If 3.2 Which interdependence did you observe? = Both

Q70 **3.2** Please explain why you selected 'both'.

____

Page Break

Q71 **3.3** Please review this clip as needed to refresh your memory.

Q72 **3.3** Interdependence definition
Patterns of interactions between individuals that can afford or constrain one's performance and shape the practice of the broader healthcare team.

Did you observe interdependence between the EM RESIDENT and anyone else in this clip?

- ○ Yes (1)
- ○ No (2)

Page Break

Display This Question:

If 3.3 Interdependence definition Patterns of interactions between individuals that can afford or co... = No

Q73 **3.3** Why did you answer 'no' to the previous question?

____

Page Break

Display This Question:

If 3.3 Interdependence definition Patterns of interactions between individuals that can afford or co... = Yes

Q74 **3.3** Which interdependence did you observe?

○ Supportive Interdependence [Triggered by a resident's lack of expertise within their scope of practice.] (1)

○ Collaborative Interdependence [Triggered by a recognition that patient care requires contributions from other team members.] (2)

○ Both (3)

Page Break

Display This Question:

If 3.3 Interdependence definition Patterns of interactions between individuals that can afford or co... = Yes

And 3.3 Which interdependence did you observe? = Supportive Interdependence [Triggered by a resident's lack of expertise within their scope of practice.]

Or 3.3 Which interdependence did you observe? = Collaborative Interdependence [Triggered by a recognition that patient care requires contributions from other team members.]

Q75 **3.3** Please explain why you selected this type of interdependence.

____

Page Break

Display This Question:

If 3.3 Which interdependence did you observe? = Both

Q76 **3.3** Please explain why you selected 'both'.

____

Page Break

Q77 **3.4** Please review this clip as needed to refresh your memory.

Q78 **3.4** <u>Interdependence definition</u>
Patterns of interactions between individuals that can afford or constrain one's performance and shape the practice of the broader healthcare team.

Did you observe interdependence between the EM RESIDENT and anyone else in this clip?

- ○ Yes (1)
- ○ No (2)

Page Break

Display This Question:

If 3.4 Interdependence definition Patterns of interactions between individuals that can afford or co... = No

Q79 **3.4** Why did you answer 'no' to the previous question?

____

Page Break

Display This Question:

If 3.4 Interdependence definition Patterns of interactions between individuals that can afford or co... = Yes

Q80 **3.4** Which interdependence did you observe?

○ Supportive Interdependence [Triggered by a resident's lack of expertise within their scope of practice.] (1)

○ Collaborative Interdependence [Triggered by a recognition that patient care requires contributions from other team members.] (2)

○ Both (3)

Page Break

Display This Question:

If 3.4 Which interdependence did you observe? = Both

Q81 **3.4** Please explain why you selected 'both'.

____

Page Break

Display This Question:

If 3.4 Which interdependence did you observe? = Supportive Interdependence [Triggered by a resident's lack of expertise within their scope of practice.]

Or 3.4 Which interdependence did you observe? = Collaborative Interdependence [Triggered by a recognition that patient care requires contributions from other team members.]

Q82 **3.4** Please explain why you selected this type of interdependence.

____

Page Break

Q83 Case 3: Overall, was the degree of interdependence appropriate based on the whole scenario?

- Yes (1)
- No (2)

Q84 Please explain why or why not.

____

Page Break

Q85 **Milestones**
What milestone rating would you give to this EM resident, based on all three videos?

Systems - Based Practice 3 : System Navigation for Patient Centered Care

- ○ Level 1: Demonstrates knowledge of care coordination. Identifies key elements for safe and effective transitions of care and hand-offs. Demonstrates knowledge of population and community health needs and disparities. (1)

- ○ Level 2: In routine clinical situations, effectively coordinates patient care integrating the roles of interprofessional teams. In routine clinical situations, enables safe and effective transitions of care/hand-offs. Identifies specific population and community health needs and inequities for their local population. (29)

- ○ Level 3: In complex clinical situations, effectively coordinates patient care by integrating the roles of the interprofessional teams. In complex clinical situations, enables safe and effective transitions of care/hand-offs. Effectively uses local resources to meet the needs of a patient population and community. (30)

- ○ Level 4: Serves as a role model, effectively coordinates patient-centered care among different disciplines and specialties. Serves as a role model, advocates for safe and effective transitions of care/hand-offs within and across health care delivery systems, including outpatient settings. Participates in changing and adapting practice to provide for the needs of specific populations. (27)

- ○ Level 5: Analyzes the process of care coordination and leads in the design and implementation of improvements. Improves quality of transitions of care within and across health care delivery systems to optimize patient outcomes. Leads innovations and advocates for populations and communities with health care inequities. (28)

Q86 Interpersonal and Communication Skills 2: Interprofessional and Team Communication

- ○ Level 1: Respectfully requests a consultation. Uses language that reflects the values all members of the health care team. Receives feedback in a respectful manner. (1)
- ○ Level 2: Clearly and concisely requests a consultation or other resources for patient care. Communicates information effectively with all health care team members. (31)
- ○ Level 3: Integrates recommendations made by various members of the health care team to optimize patient care. Engages in active listening to adapt to the communication styles of the team. Communicates concerns and provides feedback to peers and learners. (32)
- ○ Level 4: Acts as a role model for flexible communication strategies, i.e., those strategies that value input from all health care team members and that resolve conflict when needed. Uses effective communication to lead or manage health care teams. Communicates feedback and constructive criticism to superiors. (33)
- ○ Level 5: Acts as a role model for communication skills necessary to lead or manage health care teams. In complex situations, facilitates regular health care team-based feedback. (34)

Page Break
